# TEAMS AND TOOLS: ADDRESSING POLYPHARMACY USING THE ARMOR TOOL IN A RURAL NURSING CARE FACILITY

**DOI:** 10.1093/geroni/igac059.371

**Published:** 2022-12-20

**Authors:** Angela Zell, Raza Haque

**Affiliations:** Michigan State University, East Lansing, Michigan, United States; Michigan State University, East Lansing, Michigan, United States

## Abstract

Federal guidelines by the Centers for Medicare and Medicaid Services around the use of an interdisciplinary team (IDT) approach have been in place for years. However, use of evidence-based tools to address polypharmacy have not been specified. A study was conducted to evaluate the implementation of the ARMOR (Assess, Review, Minimize, Optimize, Reassess) tool to address polypharmacy in a rural nursing care facility. The tool provided a stepwise approach to a standardized process for the IDT to discuss individual resident care plans including psychotropic medications, psychosocial concerns, falls, quality of life, functional status and other factors in their weekly meetings. Surveys and a focus group were used to measure attitudes and skills of the IDT in relation to the use of the evidence-based tool in the IDT process. Data collected presents an overall positive attitude for and improvement in skills in the IDT with the use of the ARMOR tool.

